# Edge Artificial Intelligence Device in Real-Time Endoscopy for Classification of Gastric Neoplasms: Development and Validation Study

**DOI:** 10.3390/biomimetics9120783

**Published:** 2024-12-22

**Authors:** Eun Jeong Gong, Chang Seok Bang, Jae Jun Lee

**Affiliations:** 1 Department of Internal Medicine, Hallym University College of Medicine, Chuncheon 24253, Republic of Korea; gong-eun@hanmail.net; 2 Institute for Liver and Digestive Diseases, Hallym University, Chuncheon 24253, Republic of Korea; 3 Institute of New Frontier Research, Hallym University College of Medicine, Chuncheon 24253, Republic of Korea; iloveu59@hallym.or.kr; 4 Division of Big Data and Artificial Intelligence, Chuncheon Sacred Heart Hospital, Chuncheon 24253, Republic of Korea; 5 Department of Anesthesiology and Pain Medicine, Hallym University College of Medicine, Chuncheon 24253, Republic of Korea

**Keywords:** deep learning, endoscopy, gastric neoplasms, edge computing

## Abstract

Objective: We previously developed artificial intelligence (AI) diagnosis algorithms for predicting the six classes of stomach lesions. However, this required significant computational resources. The incorporation of AI into medical devices has evolved from centralized models to decentralized edge computing devices. In this study, a deep learning endoscopic image classification model was created to automatically categorize all phases of gastric carcinogenesis using an edge computing device. Design: A total of 15,910 endoscopic images were collected retrospectively and randomly assigned to train, validation, and internal-test datasets in an 8:1:1 ratio. The major outcomes were as follows: 1. lesion classification accuracy in six categories: normal/atrophy/intestinal metaplasia/dysplasia/early/advanced gastric cancer; and 2. the prospective evaluation of classification accuracy in real-world procedures. Results: The internal-test lesion-classification accuracy was 93.8% (95% confidence interval: 93.4–94.2%); precision was 88.6%, recall was 88.3%, and F1 score was 88.4%. For the prospective performance test, the established model attained an accuracy of 93.3% (91.5–95.1%). The established model’s lesion classification inference speed was 2–3 ms on GPU and 5–6 ms on CPU. The expert endoscopists reported no delays in lesion classification or any interference from the deep learning model throughout their exams. Conclusions: We established a deep learning endoscopic image classification model to automatically classify all stages of gastric carcinogenesis using an edge computing device.

## 1. Introduction

Gastric cancer remains a challenge to diagnose and treat, with early detection being crucial for improving survival rates [1]. Traditional methods for diagnosing gastric neoplasms rely heavily on endoscopic visual diagnosis followed by histopathological confirmation, processes that are subject to inter-observer variability. Advances in artificial intelligence (AI), particularly machine learning and deep learning, have begun to transform the landscape of medical imaging, offering new avenues for enhancing diagnostic accuracy and efficiency [2,3,4,5].

In recent years, the integration of AI within medical devices has progressed from centralized models, dependent on substantial computational resources, to decentralized edge computing devices [6]. Deploying AI models on edge devices is increasingly vital in healthcare applications, particularly in environments where real-time decision-making is critical, such as in endoscopic procedures for detecting gastric neoplasms. Edge computing facilitates data processing locally on devices that are directly at the point of care, rather than relying on remote servers. This approach significantly reduces the latency typically associated with sending data to a central server for analysis and then receiving results back. Such reductions are crucial in medical settings where every second counts, potentially affecting the outcome of diagnostic procedures and subsequent treatments. Moreover, processing data on edge devices enhances privacy and data security, critical considerations in healthcare where patient data sensitivity is paramount [7,8]. By minimizing the need to transmit potentially sensitive information over the network, edge AI mitigates risks related to data breaches and unauthorized access. Additionally, edge AI allows for continuous operation even in situations where internet connectivity is unreliable or unavailable, ensuring that the diagnostic process remains uninterrupted. This capability is particularly beneficial in rural or underserved areas where network issues may otherwise impede the use of advanced AI diagnostics. Consequently, the integration of AI into edge devices not only bolsters the efficiency and responsiveness of medical diagnostic tools but also broadens their applicability across varied clinical environments, making advanced care accessible to a wider population.

The principles of biomimetics—emulating natural systems to solve complex problems—offer significant insights into the development of advanced diagnostic tools such as the edge AI device proposed in this study. Nature, through millions of years of evolution, has optimized real-time, efficient, and decentralized information processing, as seen in neural networks within biological organisms. Similarly, the edge computing model mirrors these decentralized systems, enabling rapid, localized data analysis with minimal latency. This approach not only enhances the device’s operational efficiency but also ensures robustness in unpredictable environments, much like how biological systems adapt to varying stimuli. The integration of explainable AI techniques, such as attention maps, can also be viewed as biomimetic, resembling the way organisms focus on critical stimuli while filtering irrelevant information. By aligning technological advancements with the principles of biomimetics, this study underscores the potential of nature-inspired frameworks in enhancing the diagnostic capabilities of medical AI systems.

The authors previously created a deep learning-based AI model for classifying invasion depth (mucosa-confined vs. submucosa-invaded) in gastric neoplasms using endoscopic images [9]. This was followed by the creation of an artificial intelligence model for categorizing the histology of gastric lesions into six groups (normal mucosa, gastric atrophy, intestinal metaplasia, dysplasia, early gastric cancer (EGC), and advanced gastric cancer (AGC)) [10]. All of these models were developed using the no-code platform to improve model-building performance and ensure high accessibility for non-AI experts to meet clinical practice’s unmet needs. However, model deployments are another area of interest, and the goal of this study was to create and evaluate the efficacy and dependability of this edge AI device in a clinical environment.

## 2. Materials and Methods

### 2.1. Overall Study Design

**Figure 1:**
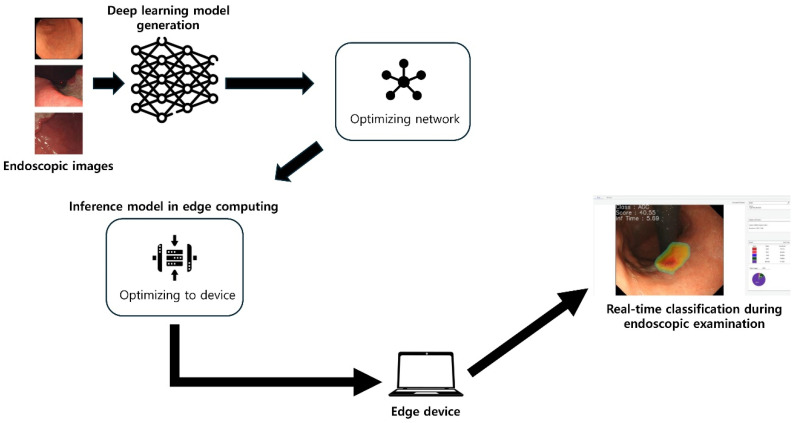
Scheme of the algorithm.

This study was approved by the institutional review board of Chuncheon Sacred Heart hospital (approval number: 2023-02-001). By using an established AI model to categorize the full steps of gastric carcinogenesis in endoscopic images on an edge computer, this study builds on earlier research [9,10]. Informed consent was not required because the data were gathered after the fact. The scheme of the algorithm is shown in Figure 1.

Initially, a large dataset of labeled endoscopic images covering a variety of stomach neoplasms was used to train the AI model. In order to verify the model’s accuracy and usability under normal operating conditions, later stages examine its performance in actual clinical scenarios. We want to lay a strong basis for the wider use of edge AI technologies in gastroenterological endoscopy by carrying out thorough developmental and validation investigations.

### 2.2. Datasets

To develop the AI-assisted lesion classification model, we extended the data collection process that was employed in previous studies [4,9,10]. We enrolled consecutive patients with any kind of gastric neoplasms found during upper gastrointestinal endoscopy and histologically confirmed at the Hallym University Chuncheon Sacred Heart hospital between 2010 and 2023. Gastric neoplasms were categorized using these three criteria: dysplasia, EGC, and AGC. Additionally, we enrolled every patient who had an upper gastrointestinal endoscopy at Hallym University Chuncheon Sacred Heart and was found to have intestinal metaplasia or atrophy. One of two categories—intestinal metaplasia or atrophy—was given to these images as a label. All enrolled images were cross-checked by two expert endoscopists (C.S.B. and E.J.G.) to minimize inter-observer variability and guarantee appropriate categorization. The contradictorily categorized images were resolved by discussion. The same process as outlined above was used to prepare the normal category, which did not exhibit intestinal metaplasia or atrophy, for the last phase. Therefore, all tumor categories were pathology-confirmed lesions; however, skilled endoscopists used visual diagnosis to classify intestinal metaplasia, atrophy, and normal mucosa. All enrolled images were cross-checked by two expert endoscopists (C.S.B. and E.J.G.) to minimize inter-observer variability and guarantee appropriate categorization. The in-hospital database was used to obtain representative endoscopic images of each patient in JPEG format, with a minimum resolution of 512 × 431 pixels. (Imaging software INFINITT Picture Archiving and Communication. System M6; INFINITT Healthcare, Seoul, Republic of Korea).

Ultimately, 15,910 endoscopic pictures were enrolled (the quantity of images and lesions were equal) and allocated at random in an 8:1:1 ratio to the train, validation, and internal-test datasets. GIF-Q260, H260, or H290 endoscopes (Olympus Optical Co., Ltd., Tokyo, Japan) were used for all internal-test and training exams, together with an endoscopic video imaging system (Evis Lucera CV-260 SL or Elite CV-290; Olympus Optical Co., Ltd., Tokyo, Japan). Table 1 describes the detailed distribution of the input images.

### 2.3. Training Dataset Preprocessing

Noise information like the patient’s name, age, gender, or identity number, as well as the date of the examination, can be found in endoscopic still-cut images [8]. Noise information was anonymized prior to training and was not stored in the training dataset. During training, various augmentation combinations or copies of augmented images were applied at random to expand the quantity of data. This led to the adoption of data augmentation techniques like adjusting hue, brightness, saturation, contrast, noise, 90° rotation, flipping included images horizontally or vertically, and image normalization with linear transformation in terms of the three RGB channels [4].

### 2.4. Creation of an AI Model

The deep learning no-code program “Neuro-X” version 3.1.1 (Neurocle Inc., Seoul, Republic of Korea) was utilized in this investigation. Based on the size of the dataset, Neuro-X provides three backbone convolutional neural network topologies to select from. Compact, normal, and heavy architectures are available, along with five different levels of hyperparameter adjustment, optimizer, decay method, batch size, epoch, and patience options. Our goal was to acquire the best performance model we could. With on-premise software, the entire deep learning model development process was accomplished by only selecting options from menus based on intuitive graphical user interfaces [4]. The AMD Ryzen Threadripper PRO 5975WX 32-Core central processing units, four RTX 3090ti graphics processing units, and 512 GB of random-access memory were all part of the training system.

### 2.5. Establishment of Edge Computing Device

The hardware system of the edge computing device optimized for environments capable of GPU acceleration and currently operational on laptop computers was prepared with the following specifications: CPU: i9-13980HX and GPU: Nvidia RTX 4090.

The software in the edge computing device enables the real-time inference of established AI models, featuring a graphical user interface that allows users to run model inference and visualize results through simple button clicks, eliminating the need for coding. The software leverages C++ as the primary programming language, selected for its efficiency in low-level hardware manipulation, thereby ensuring superior performance and speed. This established software supports three input modes: real-time camera streams, images, and video files. It offers support for a variety of file formats, including jpg, png, and bmp. The software consists of the following components: Model Input Component: imports the established deep learning model. Image Data Input Component: captures data from real-time cameras, stored images, or video files. Output Display Component: renders the inference results on the original and overlay images. Result Summary Component: displays a comprehensive summary of the outcomes, including inference speed, detection results, and probability values.

The model inference process is executed within an engine that is finely tuned for the hardware, significantly reducing inference times across various devices. The optimization techniques include the following: 1. Parallel computing, which implements parallel processing across the GPU and CPU to enhance computational performance. 2. Reduced precision, which utilizes reduced precision techniques, transitioning operations from 32-bit floating point (FP32) precision to FP16 (automatic mixed precision, AMP) to augment performance while maintaining accuracy. 3. Layer and Tensor Fusion, which fuses deep learning layers to enhance data efficiency. 4. Kernel Auto-Tuning, which adjusts GPU kernels to optimize performance for individual GPU architectures. 5. Dynamic Tensor Memory, which dynamically optimizes and recycles GPU memory to enhance resource utilization.

In addition to model inference, the system integrates libraries for image processing and parallel computing. This was established using the following resources: 1. OpenCV (Open-Source Computer Vision Library), employed for various image processing tasks, including image reading, preprocessing, and postprocessing. 2. OpenMP (Open Multi-Processing), a standard API designed for multi-threaded shared-memory parallel programming, which is employed to facilitate parallel processing, thereby optimizing image-processing tasks.

### 2.6. Outcomes of the Study

The accuracy of lesion-classification using the established models was the main result. Other performance measures included F1 score (2 precision * recall/precision + recall), precision (defined as [true positive/true positive + false positive]), and recall (defined as [true positive/true positive + false negative]).

### 2.7. Verification of Prospective Performance in Real-World Procedures

A performance verification (prospective test) was conducted prospectively, utilizing real-world procedures to guarantee the generalizability of categorization performance. Using a state-of-the-art established edge computing device, a series of upper endoscopic examinations conducted at Chuncheon Sacred Heart Hospital between January and May 2024 were examined. This AI-assisted endoscopic examination involved two skilled endoscopists, C.S.B. and E.J.G. A GIF-Q260, H260, or H290 endoscope (Olympus Optical Co., Ltd., Tokyo, Japan) and an endoscopic video imaging system (Evis Lucera CV-260 SL or Elite CV-290; Olympus Optical Co., Ltd., Tokyo, Japan) were used for all of the prospective tests. The prospective-test dataset’s comprehensive distribution is shown in Table 1.

### 2.8. Analysis of Attention Maps for Explainability

In order to identify the particular class in the provided photos, a gradient-weighted class activation map, also known as an attention map, was incorporated into the neural network layer. For every test image, an attention map was made and examined [4].

## 3. Results

### 3.1. Features of the Dataset

15,910 endoscopic images in all were collected retrospectively and split into the train, validation, and internal-test datasets at random in an 8:1:1 ratio. ‘Atrophy’ accounted for the largest percentage of the total images [23.8% (3787/15,910)], followed by ‘normal’ [27.3% (4338/15,910)] and ‘intestinal metaplasia’ [23.8% (3787/15,910)]. ‘EGC’ showed the largest percentage of neoplastic lesions [6.9% (1104/15,910)], followed by ‘dysplasia’ [6.3% (1008/15,910)] and ‘AGC’ [4.3% (679/15,910)].

For the prospective-test set, 718 unique images that weren’t in the training, validation, or internal-test datasets were collected. Following “normal” [35.9%, (258/718)] and “intestinal metaplasia” [21.9%, (156/718)], the proportion of “atrophy” was the greatest [39.1%, (281/718)]. In the neoplasm categories, “dysplasia” had the largest percentage [1.4% (10/718)], followed by “AGC” [1.0% (7/718)] and “EGC” [0.7% (5/718)]. The quantity and distribution of each category in the dataset are shown in Table 1.

### 3.2. Hyperparameters for Training When Creating an AI Model

The AI model was established using the on-premise software’s proprietary neural network structure, Adam optimizer, with a batch size of 72, epoch of 100, patience of 30, and the Cosine learning rate decay method (decay ends after 11,400 steps, initial learning rate 0.002). Categorical cross-entropy was the loss function. At epoch 34/65, the train loss was 0.1319 and the validation loss was 0.0287.

### 3.3. Performance in Internal Test

**Figure 2:**
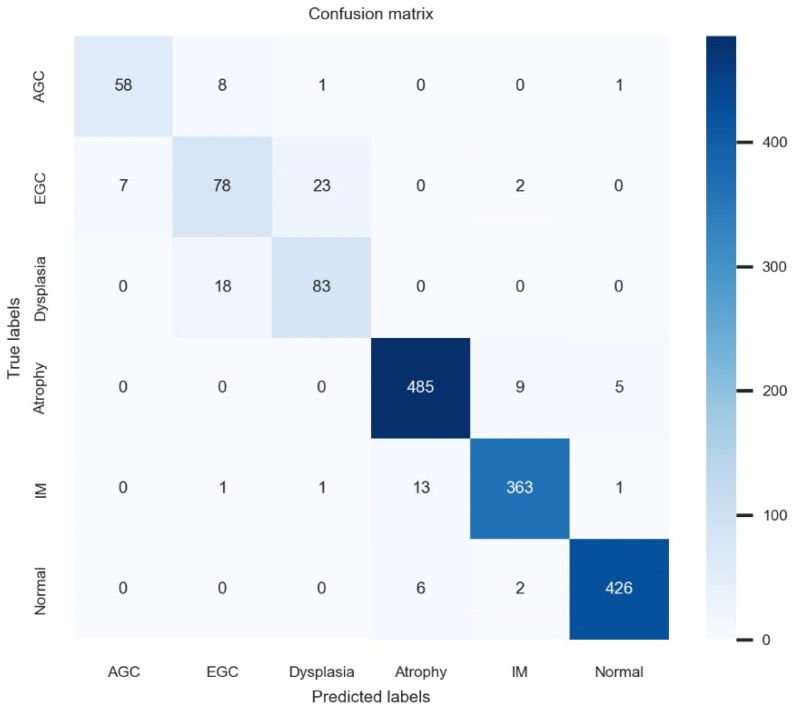
Confusion matrix for the internal test.

In an internal test, the established model’s accuracy was 93.8% (95% CI: 93.4–94.2%), precision was 88.6% (88.1–89.1%), recall was 88.3% (87.8–88.8), and its F1 score was 88.4% (87.9–88.9%). AGC had a per-class Area Under the Receiver Operating Characteristic curve, AUROC of 96.8%, EGC 94.5%, dysplasia 96.8%, atrophy 97.9%, intestinal metaplasia 98.3%, and the normal category 99.1%. The confusion matrix for internal-test performance is shown in Figure 2.

### 3.4. Prospective-Test Performance

**Figure 3:**
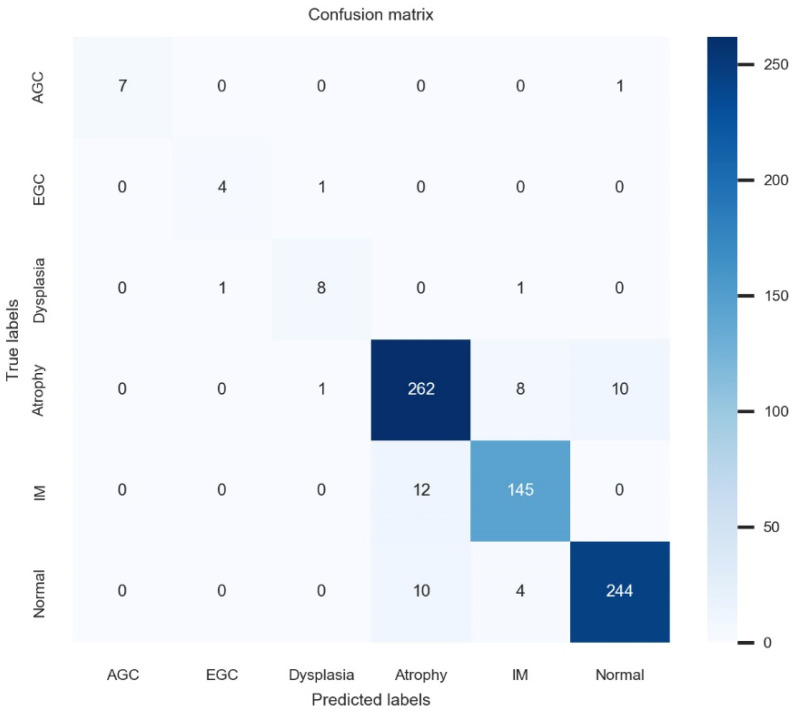
Confusion matrix for the prospective validation test.

For its prospective-test performance, the established model achieved an accuracy of 93.3% (95% CI, 91.5–95.1%), a precision of 93.2% (91.4–93.4%), a recall of 93.2% (91.4–93.4%), and a F1 score of 93.2% (91.4–93.4%). Table 2 shows the detailed performance. Figure 3 demonstrates the confusion matrix for the prospective-test performance. The inference speed for lesion classification of the established model was 2–3 ms on GPU mode and 5–6 ms on CPU mode (employing a 640 × 380 image at a batch size of 1, on hardware comprising an i9-13980HX CPU and an RTX 4080 GPU). The expert endoscopists did not experience delays in lesion classification time or feel that the deep learning model interfered with their exams. Supplementary Material S1 shows the representative case of edge device-assisted endoscopic examination. An about 3 cm sized EGC lesion was suspected at first by the AI model. However, after giving more information to the AI model, the diagnosis was changed to AGC.

### 3.5. Attention Map Analysis

**Figure 4:**
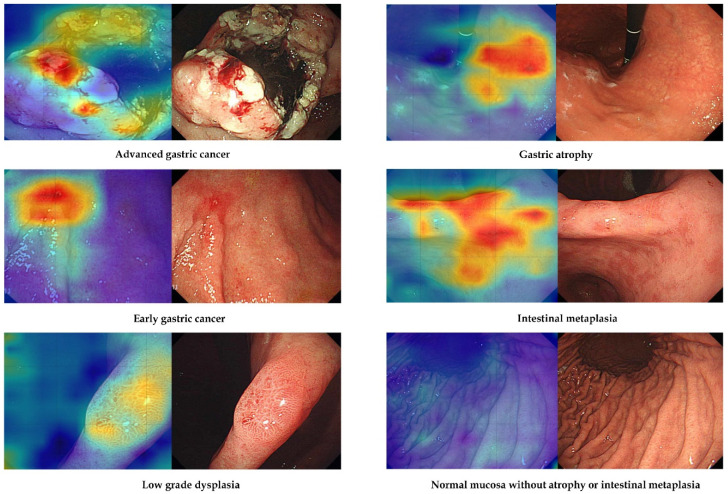
Representative images of attention map analysis.

Figure 4 shows representative cases that were correctly determined as regions of interest by the established AI model. The right panel shows that each neoplastic or preneoplastic lesion and region of interest is well matched with the AI model’s focus. Focal atrophic mucosa or intestinal metaplastic regions are also well matched with the AI model’s focus.

## 4. Discussion

In this study, we successfully developed and validated an AI-assisted endoscopic diagnostic tool as an edge computing device to classify gastric neoplasms in real-time. The results demonstrated high accuracy in both internal-test and real-world settings, confirming the tool’s potential to enhance clinical outcomes in the classification of gastric lesions. The performance of our edge AI model was found to be comparable to previous studies that used server-based approaches for traditional transfer-learning-based deep learning models [4,10,11]. As far as we know, this is the first clinical study on the establishment and test performance of AI-assisted endoscopy in an edge computing device.

The primary achievement of this study lies in the successful implementation of a deep learning model on an edge computing device for real-time endoscopic classification. With an internal-test accuracy of 93.8% and a prospective-test accuracy of 93.3%, the results underscore the reliability of our AI model in classifying various gastric neoplasms, including early and advanced gastric cancer, dysplasia, atrophy, and intestinal metaplasia. Importantly, the inference time of 2–3 ms on GPU mode and 5–6 ms on CPU mode highlights the feasibility of real-time application without any noticeable delays, which is crucial for clinical workflows that require instantaneous decision-making.

A key strength of this research is the deployment of AI on an edge computing device. Unlike traditional AI systems that require cloud servers and substantial computational resources [4,5,9,10,11], edge AI operates locally, thereby eliminating dependence on a stable network connection. This capability is particularly beneficial in clinical environments where reliable internet access may be limited, such as in rural or underserved areas. By processing data locally, edge AI not only reduces latency, which is critical during endoscopic examinations, but also ensures better privacy and security for patient data by minimizing the risk of data breaches during network transmissions [6,7]. In the context of healthcare, where the sensitivity of patient data is paramount, this feature of edge AI makes it an attractive solution.

The clinical implications of this research are significant. Real-time endoscopic classification can facilitate the early detection of gastric neoplasms, which is crucial for improving patient outcomes [1,3,4]. EGC, if detected promptly, has a markedly better prognosis compared to advanced stages. The high accuracy of our AI model, combined with the capability to function in an edge device, offers an effective tool for reducing diagnostic errors, which are often attributed to inter-observer variability among endoscopists. The AI model’s consistent performance could contribute to standardizing diagnostic accuracy across different clinicians, ultimately improving the quality of care [12,13,14,15,16,17,18,19,20].

Despite these promising results, there are limitations that must be acknowledged. One notable limitation is the generalizability of the model. The training and validation of the AI model were conducted using data collected from a single medical institution with specific endoscopic equipment (Olympus Optical Co., Ltd., Tokyo, Japan). This introduces potential biases and may limit the applicability of the model to other institutions with different types of endoscopic devices or populations with different clinical characteristics. Future studies should include datasets from multiple centers and diverse patient populations to improve the robustness and generalizability of the model [21].

Another limitation lies in the dependence on high-performance hardware. While edge computing offers significant benefits, the performance of the AI model is closely tied to the specifications of the hardware used. Our study used a high-performance edge device with GPU acceleration, which may not be feasible in all clinical settings, particularly in resource-limited environments. The need for high-end hardware could pose a barrier to the widespread adoption of this technology. Therefore, optimizing the model to work effectively on lower-spec hardware while maintaining diagnostic accuracy is a key area for future development.

Furthermore, while our model achieved high accuracy in classification, the inclusion of only static images for training means the model might not be fully optimized for live endoscopic video streams, which can include additional challenges such as motion artifacts, variable lighting conditions, and rapid changes in the field of view [22]. Although Supplementary Material S1 (real-world application movie) has shown sufficiently high performance with real-world applicability, integrating the model with various live video data and testing its robustness in such dynamic conditions is an essential step for future studies.

Building on the strengths and addressing the limitations of this study, several future research directions are proposed. First, multi-center validation studies are needed to assess the model’s performance across various settings, which would help ensure the generalizability of the findings. Such studies should include a diverse patient demographic and different types of endoscopic equipment to validate the model’s adaptability [23,24,25,26]. Second, optimizing the AI model for deployment on a wider range of edge devices, including those with limited computational power, is critical for ensuring broader applicability. Approaches such as model pruning, quantization, and knowledge distillation could be explored to reduce computational demands without significantly sacrificing accuracy. These optimizations could facilitate the adoption of AI-assisted endoscopic diagnostics in clinics that lack high-performance hardware. Another important avenue for future research is the development of a self-learning capability for the AI model. By enabling continuous learning from new data collected during clinical use, the model could adapt and improve its diagnostic performance over time. Such an approach would make the AI tool more robust in accommodating variability in endoscopic appearances, patient demographics, and operator techniques. Lastly, incorporating advanced visualization techniques, such as more interpretable attention maps, could help endoscopists better understand the AI model’s decision-making process [27]. In the model we developed, there is a possibility that the real-time visible attention map may interfere with the inspector’s view. Therefore, it is speculated that simplification to a detection box that does not interfere with the observation of lesions or adjusting the tone of the attention map is necessary, and the constantly changing classification answer needs to be more intuitive. This could increase clinician confidence in using AI as a diagnostic aid, fostering trust and acceptance of the technology in routine practice. Providing clear visual cues regarding why certain areas are identified as neoplastic would enhance the explainability of the model, which is a critical aspect of AI integration in healthcare [28,29,30,31].

In conclusion, this study demonstrates the feasibility of deploying a deep learning diagnostic model for gastric neoplasms on an edge computing device. The high accuracy and real-time performance of the model, combined with the inherent benefits of edge computing, make it a useful tool for enhancing the diagnostic capabilities of endoscopic procedures. However, further work is needed to validate the model across diverse settings, optimize its use on lower-spec devices, and integrate advanced features such as self-learning and explainable AI. By addressing these challenges, edge AI could play a pivotal role in transforming the landscape of gastroenterological diagnostics, making high-quality care more accessible and consistent across different clinical environments.

## Figures and Tables

**Table 1:** Data distribution for establishment and test of computer-aided diagnosis model.

	Whole Dataset	Training Dataset	Validation Dataset	Internal-Test Dataset	Prospective Real-Clinic Evaluation Dataset
Overall	15,910	12,728	1591	1591	718
Advanced gastric cancer	679 (4.3%)	543	68	68	7 (1.0%)
Early gastric cancer	1104 (6.9%)	884	110	110	5 (0.7%)
Dysplasia	1008 (6.3%)	806	101	101	10 (1.4%)
Atrophy	4994 (31.4%)	3996	499	499	281 (39.1%)
Intestinal metaplasia	3787 (23.8%)	3029	379	379	157 (21.9%)
Normal	4338 (27.3%)	3470	434	434	258 (35.9%)

**Table 2:** Performance in the prospective test.

	Precision (95% Confidence Interval)	Recall (95% Confidence Interval)	F1 score (95% Confidence Interval)
Overall	93.2% (91.4–93.4%)	93.2% (91.4–93.4%)	93.2% (91.4–93.4%)
Advanced gastric cancer	99.9% (97.7–99.9%)	87.5% (64.6–99.9%)	93.3% (76.0–99.9%)
Early gastric cancer	80.0% (48.0–99.9%)	80.0% (48.0–99.9%)	80.0% (48.0–99.9%)
Dysplasia	80.0% (56.4–99.9%)	80.0% (48.0–99.9%)	80.0% (48.0–99.9%)
Atrophy	92.3% (89.5–95.1%)	93.2% (90.4–96.0%)	92.7% (89.8–95.6%)
Intestinal metaplasia	91.7% (87.6–95.8%)	92.4% (88.4–96.4%)	92.0% (87.9–96.1%)
Normal	95.6% (93.1–98.1%)	94.6% (91.9–97.3%)	95.0% (92.4–97.6%)

## Supplementary Materials

The following supporting information can be downloaded at: https://www.mdpi.com/article/10.3390/biomimetics9120783/s1, Supplementary Material S1: real-world application movie.
